# Evidence for interactions between the mitochondrial import apparatus and respiratory chain complexes via Tim21-like proteins in Arabidopsis

**DOI:** 10.3389/fpls.2014.00082

**Published:** 2014-03-11

**Authors:** Monika W. Murcha, Szymon Kubiszewski-Jakubiak, Yan Wang, James Whelan

**Affiliations:** ^1^ARC Centre of Excellence in Plant Energy Biology, The University of Western AustraliaPerth, WA, Australia; ^2^Department of Botany, ARC Centre of Excellence in Plant Energy Biology, School of Life Sciences, La Trobe UniversityBundoora, VIC, Australia

**Keywords:** protein import, Arabidopsis, mitochondrial biogenesis, Tim21, respiratory complexes

## Abstract

The mitochondrial import machinery and the respiratory chain complexes of the inner membrane are highly interdependent for the efficient import and assembly of nuclear encoded respiratory chain components and for the generation of a proton motive force essential for protein translocation into or across the inner membrane. In plant and non-plant systems functional, physical, and evolutionary associations have been observed between proteins of the respiratory chain and protein import apparatus. Here we identify two novel Tim21-like proteins encoded by At2g40800 and At3g56430 that are imported into the mitochondrial inner membrane. We propose that Tim21-like proteins may associate with respiratory chain Complex I, III, in addition to the TIM17:23 translocase of the inner membrane. These results are discussed further with regards to the regulation of mitochondrial activity and biogenesis.

## Introduction

Mitochondria are membrane bound organelles that play essential roles in metabolism, energy production, and biosynthesis of a variety of compounds in almost all eukaryotic cells. They are endosymbiotic in origin, and over time the majority of genes in the endosymbiont were lost or transferred to the host nucleus (Gray et al., 2001). Thus, the majority of the 1000 or more proteins located in the mitochondria are encoded by nuclear genes, translated in the cytosol, and imported via specialized components and protein complexes on the outer, intermembrane space, and inner membrane (Balsera et al., 2009).

The core components and mechanisms involved in mitochondrial protein import and assembly were established in the earliest eukaryotes with ancient origins of many components of the protein import apparatus (Albrecht et al., 2010; Delage et al., 2011; Hewitt et al., 2011). This is evidenced by the presence of many core and central components, such as the translocase of the outer membrane (TOM), sorting and assembly machinery (SAM), and the translocases of the inner membrane (TIM), in stramenophiles and hydrosomes (Lithgow and Schneider, 2010; Delage et al., 2011; Hewitt et al., 2011; Heinz and Lithgow, 2013). These ancient fundamental transporters originating from bacterial ancestors, exhibit a high level of conservation across the eukaryotic kingdoms from unicellular eukaryotes to plants, present in nearly all systems studied to date (Liu et al., 2011). Nevertheless, throughout the course of evolution, major adaptions have occurred to deal with the complexity of multi-cellular environments or in the case of plants the acquisition of an additional organelle, the plastid, and the adaptation to terrestrial environments. Therefore, the selective pressure to maintain protein targeting specificity and efficiency has led to the evolution of unique and specialized features for the regulation of mitochondrial biogenesis in plants (Duncan et al., 2013; Murcha et al., 2014).

Examples of the divergence and/or acquisition of functions within the plant mitochondrial machinery exist within nearly all protein complexes and compartments. Firstly, the cytosolic precursor protein is recognized by the outer membrane receptor Tom20, a cytosolic facing receptor anchored to the outer membrane. Plant Tom20 is evolutionary distinct from yeast Tom20 being identified via biochemical means (Werhahn et al., 2001), rather than homology, and is anchored to the outer membrane via its C-terminus. This is the opposite of what is observed in yeast and mammalian models with Tom20 being anchored via the N-terminal domain and thus plant Tom20 provides an example of functional convergence of two distinct genes (Perry et al., 2008; Rimmer et al., 2011). Several examples of disparities also exist on the inner mitochondrial membrane, such as with the TIM17:23 translocase channel, responsible for the import of the majority of the matrix located and certain inner membrane proteins (Rehling et al., 2001). A unique feature of plants is that it is Tim17 that links the inner and outer membranes, and not Tim23 as observed in yeast (Donzeau et al., 2000; Murcha et al., 2005a).

Whilst there are several unique features within the plant mitochondrial import apparatus it is interesting that to date only one plant specific import component has been identified, OM64 (outer membrane protein 64) (Chew et al., 2004; Lister et al., 2007). OM64 is actually paralog to a chloroplast import component TOC64 (translocase of the outer envelope of chloroplasts) but is localized to the mitochondrial outer membrane and shown to import a subset of precursor proteins (Chew et al., 2004; Lister et al., 2007). Whilst deletion of OM64 does not cause deleterious effects, it can compensate to some degree for the loss of plant Tom20, as a quadruple mutant of all three *tom20* isoforms and *om64* is not viable (Lister et al., 2007; Duncan et al., 2013).

The functional divergence of the plant mitochondrial import machinery is partly due to the large expansion of gene family members, a good example is the Preprotein and Amino Acid Transporters family (PRAT) that constitute the inner membrane translocases (Rassow et al., 1999). This family of transporters originate from a single eubacterial ancestor and in yeast comprises the inner membrane translocases Tim17, Tim23, and Tim22 (Rassow et al., 1999). Conversely, in Arabidopsis this gene family has expanded to 17 members, including the inner membrane translocases along with the chloroplast outer envelope proteins (OEP16) with multiple isoforms encoding each transporter (Murcha et al., 2007; Pudelski et al., 2010). Sub-functionalization can also be observed within PRAT sub-groups, such as the TIM17's. Of the three isoforms that encode Tim17 in Arabidopsis, AtTim17-1, AtTim17-2 and AtTim17-3, isoforms 1 and 2 contain the C-terminal extension that was shown to link both membranes, whilst the third, is significantly shorter, and most similar to yeast Tim17 (Murcha et al., 2003, 2005a). Complementation studies further revealed that AtTim17-2 could only complement the yeast deletion strain when the carboxy-terminal extension was removed (Murcha et al., 2003). Additionally, each isoform of Tim17 exhibits differential expression profiles throughout development and in response to stress providing insights into their functional roles (Lister et al., 2004; Duncan et al., 2013).

Plant mitochondria lack direct orthologs to several yeast mitochondrial import components, such as Tim12, Tim54, Tim18, and Tom70 (Murcha et al., 2014), all of which are either essential or integral for yeast growth and viability (Hines et al., 1990; Kerscher et al., 1997, 2000; Sirrenberg et al., 1998). This emphasizes the likelihood that specialized novel proteins may evolve and only be present in a lineage specific manner.

The complexity of the mitochondrial import apparatus is further exemplified by cases of interactions between proteins of the import apparatus and components of the respiratory chain. The first example identified was the cytochrome bc_1_/MPP (mitochondrial processing peptidase) complex. In plants the cytochrome bc_1_ complex is bi-functional playing both roles in electron transfer and in the proteolytic removal of mitochondrial targeting sequences (Braun et al., 1992; Glaser et al., 1994). Further examples now exist in both plant and non-plant species of proteins which can have either a dual-function in respiration and protein import or a dual-location, i.e., associated with the protein import machinery and of the respiratory chain (Van der Laan et al., 2006; Saddar et al., 2008; Gebert et al., 2011; Kulawiak et al., 2012; Wang et al., 2012). Such inter-functional interactions are thought to be beneficial for the efficient import and assembly of respiratory chain subunits, and to maintain a membrane potential required via the inner membrane translocases (Kulawiak et al., 2012). Tim23 has also been shown to interact with the respiratory apparatus and is located within both respiratory Complex I and TIM17:23 in Arabidopsis (Wang et al., 2012). Furthermore, the Complex I subunit B14.7 (Meyer et al., 2008; Klodmann et al., 2010) is also associated with the TIM17:23 complex (Wang et al., 2012). It was proposed that the dual-location of protein in two complexes might be a mechanism to co-ordinate mitochondrial activity and biogenesis due to the inverse relationship between the abundance of Tim23 and the abundance of Complex I (Murcha et al., 2012; Wang et al., 2012).

Tim21 is an interesting example of dynamic interactions, initially identified as a subunit of the TIM17:23 translocation complex has also been shown to interact with the respiratory apparatus but also the TOM complex of the outer membrane. In yeast, Tim21's association with TIM17:23 forms the sorting and organization translocase (SORT) complex along with accessory protein Tim50 (Van der Laan et al., 2006). This dynamic configuration of TIM17:23 promotes the tethering of Tim21 to the outer membrane translocation pore and initiates the insertion of proteins into the inner membrane (Chacinska et al., 2005; Mokranjac et al., 2005). Further characterization of yeast Tim21 revealed its physical association with both components of the TIM17:23 complex respiratory subunits of complex III and IV (cytochrome c1, Rieske Fe/S, and cox4). In addition, it was shown to have a direct role in the import and insertion of proteins into the inner membrane and not in the translocation of matrix located proteins (Van der Laan et al., 2006).

In Arabidopsis, one gene encodes for Tim21 termed AtTim21 (At4g00026) (Carrie et al., 2010; Murcha et al., 2014). Similarly as observed with yeast Tim21, AtTim21 was shown to interact with TIM17:23 complex and Complex III (Wang et al., 2012). Furthermore, deletion of AtTim21 results in early seedling lethality (Hamasaki et al., 2012), which is in contrast to its non-essential nature in yeast (Chacinska et al., 2005). Moreover, an over-expression line of AtTim21 exhibited increased cell numbers, cell size and ATP production, whilst the transcript abundance of complex III, IV, and ATP synthase subunits was also up-regulated (Hamasaki et al., 2012). Therefore, both studies support the premise that AtTim21 may also be involved in the import and biogenesis of respiratory chain components.

In this study, we identify two additional Tim21-like proteins in Arabidopsis encoded by At2g40800 and At3g56430. Whereas they contain the conserved Tim21 protein domain (conserved in all Tim21 proteins), they are phylogenetically distinct from the Tim21 family and thus are termed as Tim21-like. Tim21-like proteins appear to be plant specific, originating in green algae and are identified in almost all angiosperms tested. As with AtTim21, AtTim21-like proteins are also targeted to the mitochondria and biochemical characterization suggests that AtTim21-like 1 and 2 have the ability to interact with the TIM17:23 complex, and Complex I and III.

## Materials and methods

### Phylogenetic analyses

AtTim21-like 1 (At2g40800) and AtTim21-like 2 (At3g56430) were selected for investigation due to the presence of the Tim21 protein domain (PF08294) recognized in the Conserved Domains Database (Marchler-Bauer et al., 2011). Orthologs to AtTim21 and AtTim21-like 1 and 2 were identified by sequence homology using BLASTN (Altschul and Koonin, 1998) within each respective non-plant species databases and using Phytozome 9.1 (Goodstein et al., 2012) for all plant species. Orthologs were manually curated to exclude proteins with a similarity threshold above 1e^−10^. Alignments were performed using ClustalOmega (Sievers et al., 2011) (www.ebi.ac.uk) and the percentage identity and similarity scores were determined using MatGAT 2 (Campanella et al., 2003). Transmembrane domains were identified using TMHMM (Krogh et al., 2001) and targeting signals predicted using MitoProt II (Claros, 1995). The phylogenetic tree was analyzed and drawn using MEGA 5.2.2 (Tamura et al., 2011) using the maximum likelihood tree method and the Jones-Thornton-Taylor model after 1000 replications.

### cDNA clones

Full-length cDNA was amplified using gene-specific primers flanked by Gateway recombination cassettes (Supplemental Table 1) and cloned into C-terminal GFP fusion vectors (Carrie et al., 2009) for GFP localization or pDEST14 (Invitrogen), for *in vitro* transcription and translation under a T7 promoter.

### Plant material and mitochondrial isolation

Mitochondria were isolated from 14-d old plate-grown Col-0 plants. Seeds were sterilized with chlorine gas and sown on MS media followed by 48 h stratification at 4°C and grown with a light intensity of 80 nmol quanta m^−2^ s^−1^ in a 16-h photoperiod.

### GFP localization assays

Biolistic co-transformation of GFP and mt Cherry fusion vector (Nelson et al., 2007) was carried out on 5-d old Arabidopsis cell suspensions as previously described (Carrie et al., 2009). 5 μ g of GFP and mt Cherry plasmids were co-precipitated onto gold particles and bombarded using the PDS-1000/He biolistic transformation system (Bio-Rad). GFP and mt Cherry expression was visualized and captured at 100X magnification using the BX61 Olympus microscope at 460/480 nM (GFP) and 570–625 nm (mt Cherry).

### Protein import and BN-page analysis

Precursor proteins were radiolabeled using the rabbit reticulocyte T_*N*_T *in vitro* transcription/translation kit (Promega) accordingly to manufacturer's instructions. In small-scale *in vitro* import experiments, 100 ug of mitochondria were incubated in 90 μ l of ice-cold import master mix (0.3 M sucrose, 50 mM KCl, 10 mM MOPS, 5 mM KH_2_PO_4_, 0.1% [w/v] BSA, 1 mM MgCl_2_, 1 mM Met, 0.2 mM ADP, 0.75 mM ATP, 5 mM succinate, 5 mM DTT, 1 mM GTP, and 1 mM NADH, pH 7.5) with or without the addition of 1 μ M valinomycin for 3 min on ice. Following incubation, 10 μ l of [^35^S]-labeled precursor protein was added and the import reaction was initiated by incubation at 26°C at 350 rpm. Following import, tubes were moved to ice and 3.2 μ g of Proteinase K was added and incubated for a further 30 min. Proteolysis was inhibited by adding 1 μ l of 100 mM PMSF. Reactions were centrifuged (20,000 *x* g, 3 min at 4C), supernatant was discarded and pellets were re-suspended in sample buffer and analyzed by SDS-PAGE. To investigate the intra-mitochondrial location of precursor proteins, mitoplasts were prepared following the import reaction and prior to the addition of PK, as described previously (Murcha et al., 2005b). For large-scale *in vitro* import experiments, for analysis by BN-PAGE, 250 μ g of mitochondria were incubated in 360 μ l of the import master mix with 50 μ l of [35S]-labeled precursor protein. The experiments were performed as above (without the valinomycin or PK treatments) at 10, 20, and 40 min incubations and analyzed by BN-PAGE as described below.

### BN-page analysis

Mitochondrial complexes were solubilized in 5% (v/v) digitonin and separated by BN-PAGE as described previously (Meyer et al., 2009). Gels were stained with Coomassie Brilliant Blue, dried, and radiolabeled proteins were detected as outlined previously (Murcha et al., 1999).

### Yeast 2-hybrid assays

Coding regions of AtTim21-like 1, AtTim21-like 2, Tim44-2, AtTim50, AtTim22, AtTom5, AtTom9, and AtRISP, were amplified and cloned into both pGADT7 and pGBKT7 (Clontech) using primers containing restriction appropriate restriction sites at N- and C-terminus (Supplemental Table 1). Yeast vectors containing, AtTim21, AtTim17-2 AtTim23-2, AtB14.7, AtCyc1-1, and AtMPPα were used as described previously (Wang et al., 2012). The yeast 2-Hybrid screen was carried out by transforming bait (pGBKT7) and prey (pGADT7) vectors into mating compatible yeast strains, Y187, and AH109 respectively, and subsequent mating overnight. Diploid strains were plated onto selection media DDO (-Leu -Trp) for diploid selection, and QDO (-Leu -Trp -His -Ade) for indication of positive protein-protein interactions. pGBK 53 and pGAD SV40 was mated as a positive control. The plates were incubated for 5 days at 30°C.

### Accession numbers

Sequence data from this article can be found in the Arabidopsis Genome Initiative under the following accession numbers: AtTim21 (At4g00026), AtTim21-like 1 (At2g40800), AtTim21-like 2 (At3g56430), AtTim50 (At1g55900), AtTim17-2 (At2g37410), AtTim23-2 (At1g72750), AtB14.7 (At2g42210), AtTim22 (At3g10110/At1g18320), AtTim44-2 (At2g36070), AtRISP (At5g13430), AtCyc1-1 (At3g27240), AtTom5 (At5g08040), AtTom9 (At5g43970), MPPα (At3g16480), Cytochrome bd ubiquinol oxidase (At4g32470), and CAL1 (At5g63510).

## Results

**Figure 1:**
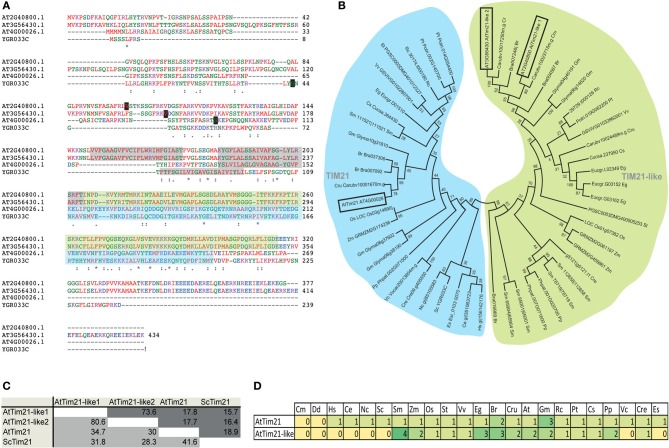
**Bioinformatic analysis of Tim21 and Tim21-like proteins from Arabidopsis. (A)** Protein alignments of Tim21 from yeast (*Saccharomyces cerevisiae*, YGR033C), AtTim21 (At4g00026) and AtTim21-like 1 and 2 proteins from Arabidopsis (At2g40800 and At3g56430). The predicted mitochondrial processing sites are boxed in black and the predicted transmembrane domains are shaded in gray. The conserved Tim21 domain is shaded in blue and green. **(B)** Protein similarity and identity scores of ScTim21, AtTim21, and AtTim21-like 1 and 2. Similarity scores are shaded in light gray and identity scores are shaded in dark gray. **(C)** Phylogenetic analysis of all Tim21 and Tim21-like proteins from 24 species, the Tim21 clade is shaded in blue, and the Tim21-like clade is shaded in green. **(D)** The number of genes encoding Tim21 and Tim21-like proteins from each species. Abbreviations: Cm, *Cyanidioschyzon merolae*; Dd, *Dictyostelium discoideum*; Hs, *Homo sapiens*; Ce, *Caenorhabditis elegans*; Sc, *Saccharomyces cerevisiae*; Sm, *Selaginella moellendorffii*; Zm, *Zea mays*; Os, *Oryza sativa*; St, *Solanum tuberosum*; Vv, *Vitis vinifera*; Eg, *Eucalyptus grandis*; Br, *Brassica rapa*; Cru, *Capsella rubella*; At, *Arabidopsis thaliana*; Gm, *Glycine max*; Rc, *Ricinus communis*; Pt, *Populus trichocarpa*; Cs, *Cucumis sativus*; Pp, *Physcomitrella patens*; Vc, *Volvox carteri*; Cre, *Chlamydomonas reinhardtii*; Es, *Ectocarpus siliculosus*. An ^*^ (asterisk) indicates positions which have a single, fully conserved residue. A : (Colon) indicates conservation between groups of strongly similar properties-scoring > 0.5 in the Gonnet PAM 250 matrix. A . (Period) indicates conservation between groups of weakly similar properties-scoring ≤ 0.5 in the Gonnet PAM 250 matrix.

Analysis of all Tim21 domain-containing proteins in Arabidopsis identifies AtTim21 (At4g00026) as the closest ortholog by sequence similarity and identity to yeast Tim21 (YGR033C). Two additional Tim21 domain containing proteins are also identified via the Conserved Domain Database (Marchler-Bauer et al., 2011), termed AtTim21-like 1 and AtTim21-like 2 encoded by At2g40800 and At3g56430 respectively (Figure 1A). Both are predicted to be significantly larger proteins of 414 and 434 aa compared to AtTim21 (269 aa) and ScTim21 (239 aa) and predicted to contain N-terminal mitochondrial targeting signals (Figure 1A). AtTim21-like 1 and 2 show low percentage similarity and identity scores of around 28–32% to ScTim21 and AtTim21 (Figure 1B), they are predicted to contain similar membrane spanning regions (shaded in gray) in the N-terminal portion and contain the consensus Tim21 superfamily motif (Pfam08294) as shaded in blue and green.

Phylogenetic analysis of Tim21 and Tim21-like proteins was carried out from 24 species ranging from yeast, fungi, algae (red, green and brown), and plant species representative of each evolutionary clade from *Physcomitrella patens* to *Eucalyptus grandis* (Figure 1C). Isoforms of AtTim21 and AtTim21-like proteins branch distinctly from each other suggesting that the may have diverged early as distinct lineages (Figure 1C). Tim21 proteins were identified in all species with the exception of *Cyanidioschyzon merolae* and *Dictyostelium discoideum.* Tim21-like proteins could only be identified in plants species, including the evolutionary precursors, green algae (*Chlamydomonas reinhardtii*), moss *(Physcomitrella patens)*, and spikemoss *(Selaginella moellendorffii)* (Figure 1D).

### AtTim21-like proteins are located in the mitochondria

**Figure 2:**
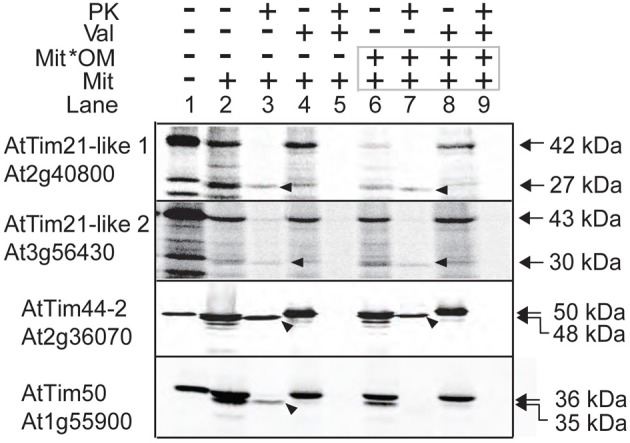
***In vitro* localization of AtTim21-like precursor proteins into isolated Arabidopsis mitochondria.** Radiolabeled precursor proteins incubated with isolated mitochondria under conditions that support import. Subsequently, mitochondria were treated with PK before or after rupture of the outer membrane via osmotic shock. Lane 1, precursor protein alone, lane 2, precursor protein incubated with mitochondria, lane 3, as lane 2 except with the addition of PK. Lane 4 and 5, as lane 2 and 3 except with the addition of valinomycin prior to import, lane 6–9, as lanes 2–5 except with rupture of the outer membrane following import and prior to PK treatment. The apparent molecular weights of the precursor and mature proteins are indicated on the right. Arrows indicate the mature processed protein. Abbreviations: Mit, mitochondria; Val, valinomycin; Mit^*^OM, mitoplasts; PK, Proteinase K.

Mitochondrial targeting predictions predict both isoforms of AtTim21-like 1 and AtTim21-like 2 to contain N-Terminal mitochondrial targeting peptides of 100 and 144 amino acids respectively. Mitochondrial import ability was tested using *in vitro* import assays of radiolabeled AtTim21-like 1 and AtTim21-like 2 into isolated Arabidopsis mitochondria. Translation of AtTim21-like 1 and AtTim21-like 2, produced radiolabeled proteins of an apparent molecular weight of 37 and 43 kDa respectively, compared to AtTim21 that has an apparent molecular mass of 27 kDa (Wang et al., 2012) Import of precursor protein into isolated Arabidopsis mitochondria produced a band of lower molecular mass of 27 and 30 kDa for AtTim21-like 1 and AtTim21-like 2, suggesting that upon import the predicted targeting peptide was cleaved (Figure 2, lanes 2 and 3). As this mature protein band was not produced in the presence of valinomycin and is Proteinase K protected, it indicates that these mature proteins are a result of import and processing in mitochondria. The mature radiolabeled bands of AtTim21-like 1 and AtTim21-like 2 were resistant to Proteinase K digestion suggesting that the mature protein is protected within the inner membrane or matrix (Figure 2, lanes 6–9). Radiolabeled AtTim21, AtTim44-2, and AtTim50, known components of the TIM17:23 translocase were used as controls. As expected mature AtTim44-2 is insensitive to protease digestion as it is located in the inner membrane matrix facing side of TIM17:23, whilst AtTim50 also located in the inner membrane but with a large portion facing the inter membrane space was largely susceptible to PK degradation (Figure 2).

**Figure 3:**
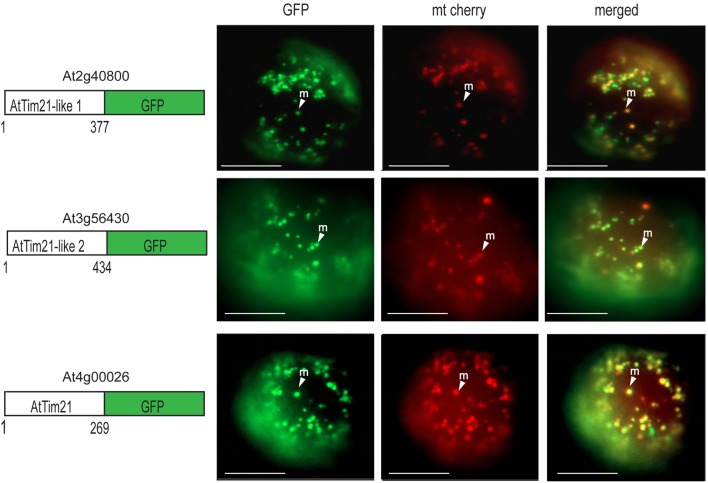
**Subcellular localizations of Arabidopsis Tim21-like proteins.** GFP was fused to the carboxy-terminus of AtTim21 (At4g00026), AtTim21-like 1 (At2g40800), and AtTim21-like 2 (At3g56430). Targeting was analyzed in Arabidopsis cell suspension along with a mitochondrial cherry control. The location of the GFP and the number of amino acids for each construct are drawn. M, mitochondria; Scale bar indicates 10 μm.

GFP targeting assays were also carried out to determine AtTim21-like 1 and AtTim21-like 2 targeting ability *in vivo.* Biolistic transformation was carried out using AtTim21-like 1 and AtTim21-like 2 constructs fused to GFP at the C-terminus. AtTim21-like 1 and AtTim21-like 2 were able to target GFP to the mitochondria (Figure 3). A mitochondrial control (mt cherry) was co-transformed as a mitochondrial control (Nelson et al., 2007). It should be noted that additional GFP signal is evident (mostly with AtTim21-like 1) that does not align to mt Cherry signal, which we suggest, results from heterogeneity in the mitochondrial population.

### AtTim21-like proteins associate with the protein import machinery and respiratory complexes

**Figure 4:**
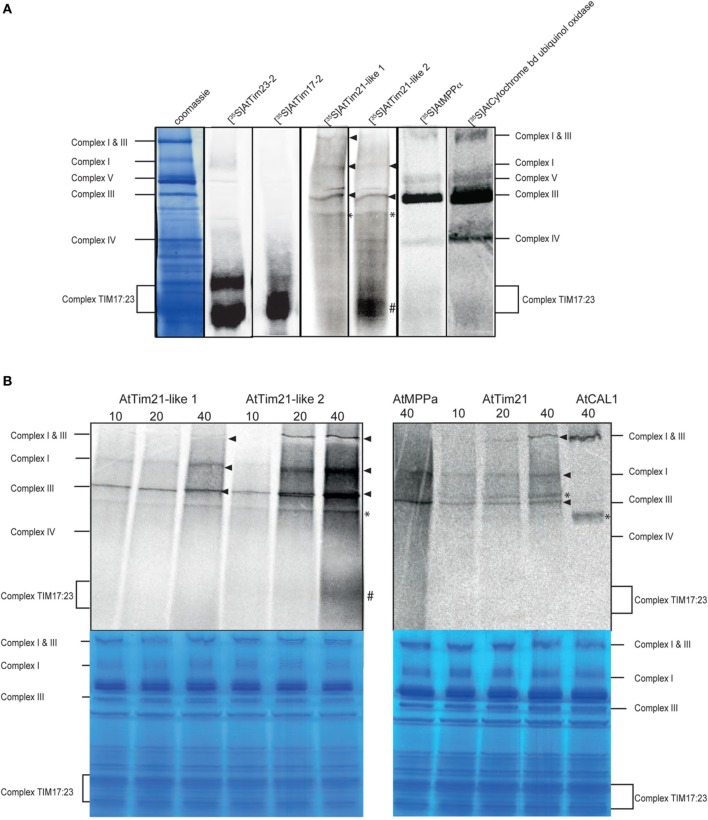
**Association of AtTim21-like proteins into mitochondrial protein complexes. (A)** Radiolabeled precursor protein was incubated with isolated Arabidopsis mitochondria for 40 min and separated by BN-PAGE. The positions of the respiratory complexes and the TIM17:23 complex are indicated. **(B)** BN-PAGE of time-course analysis (min) of AtTim21-like 1, 2 and AtTim21 import into isolated mitochondria. The incorporation of radiolabeled proteins into respiratory chain complexes is indicated by the arrow. Incorporation of radiolabeled protein into TIM17:23 complex is indicated by # and into unknown protein complexes indicated by ^*^. Corresponding coomassie stained gels are shown.

To determine the integration of AtTim21-like 1 and AtTim21-like 2 proteins within mitochondrial protein complexes, a large-scale import assay using radiolabeled AtTim21-like 1 and AtTim21-like 2 was carried out followed by BN-PAGE analysis. Import and assembly of AtTim21-like 1, AtTim21-like 2 and AtTim21 was evident within respiratory Complex III, along with Complex III subunits, MPPα (At1g51980) and ubiquinol-cytochrome C reductase (Figure 4A) (indicated by the arrow). Weak incorporation was also evident in smaller molecular weight complexes (indicated by ^*^). Incorporation of radiolabeled AtTim21-2 was also evident at a small complex at ~100 kDa, similar to the complex TIM17:23, as evidenced by the import and assembly of radiolabeled AtTim17-2 and AtTim23-2 proteins.

To confirm the incorporation of AtTim21-like 1 and AtTim21-like 2 within protein complexes was a result of specific import and accumulation, a time-course assay was carried out (Figure 4B). The incorporation of AtTim21-like 1 and AtTim21-like 2 within Complex III occurred in a time dependent manner (indicated by the arrow). Incorporation of both precursors was also evident within the monomeric form of Complex I and the supercomplex Complex I and III (Figure 4B). The import of AtTim21 was also carried out in a similar manner and similar incorporation was observed within Complex I, Complex III, and Complex I and III (Figure 4B). Time dependent labeling was also observed to unknown protein complexes (indicated by ^*^) and incorporation within the TIM17:23 complex was only observed with AtTim21-like 2 (Figure 4B) in a time dependent manner. Complex I subunit, gamma carbonic anhydrase like 1 (CAL1), was used as a Complex I control that exhibited incorporation within Complex I and III and weaker labeling within a smaller unknown complex (Figure 4B). The alpha subunit of MPP was used as a Complex III control showing incorporation into Complex III, along with weak labeling at the position of Complex I (Figure 4B).

**Figure 5:**
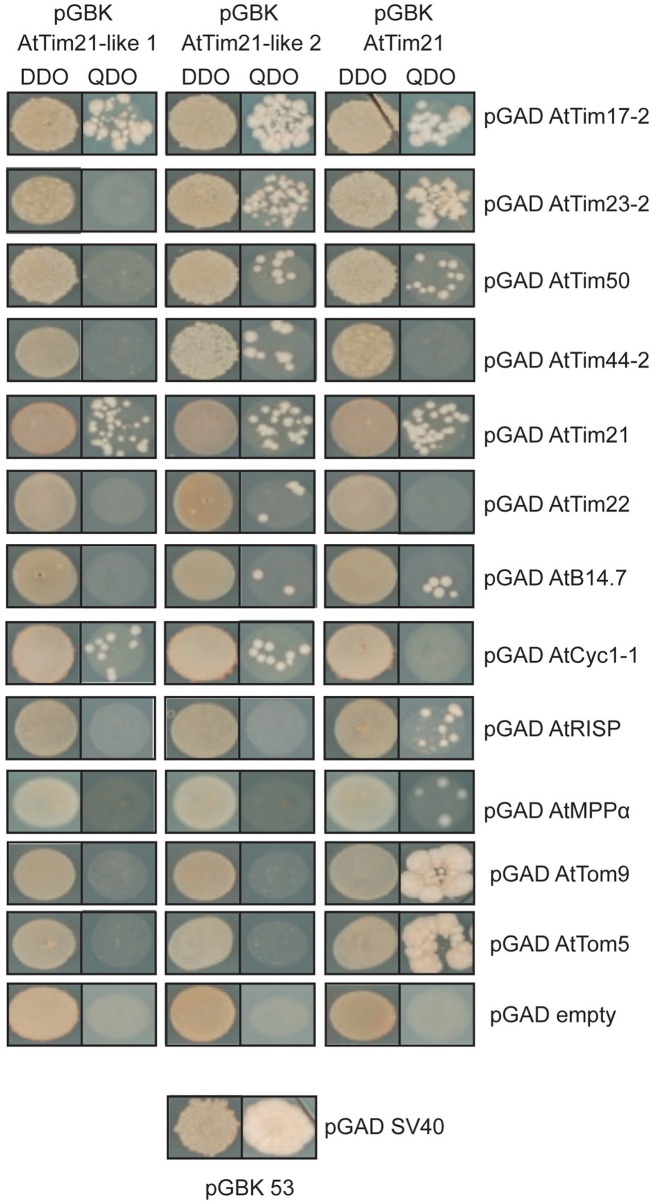
**Interactions between AtTim21-like 1, AtTim21-like 2 and components of the respiratory chain and import apparatus.** Yeast 2-Hybrid interactions indicate a positive interaction between AtTim21-like 1 and 2 and AtTim21 and various components of the mitochondrial import apparatus (AtTim17-2, AtTim23-2, AtTim50, AtTim44-2, AtTim22), Complex I (AtB14.7), Complex III (AtCyc1-1, AtRISP, AtMPPα). AtTim21 was also able to interact with TOM subunits, AtTom9, and AtTom5. Positive diploids are indicated by growth on Double Drop out media (DDO) and protein-protein interactions are indicated by growth of diploid strains on Quadruple Drop Out media (QDO). No interactions of pGBK AtTim21-like 1 and 2 and pGBK AtTim21 were observed against the pGAD empty vector. SV40 and p53 was used as a positive control.

To further confirm the interactions of AtTim21-like 1 and AtTim21-like 2 with subunits of TIM17:23 complex and respiratory chain complexes, yeast 2-Hybrid assays were carried out (Figure 5). AtTim21-like 1 and AtTim21-like 2 were cloned into the bait vector and transformed into Y187 yeast strain were mated against import components and respiratory subunits cloned into the prey vector and transformed into a mating compatible strain (AH109). The mated strains were plated out on DDO (double drop out) media to select for diploids and QDO (quadruple drop out) to determine protein-protein interactions. Positive interactions were observed between AtTim21-like 1 and 2 and AtTim21, with TIM17:23 import component, AtTim17-2. Both AtTim21-like 2 and AtTim21 interacted with AtTim23-2 and its accessory protein AtTim50. AtTim21-like 2 showed a positive interaction with AtTim44-2 and all proteins exhibited an interaction with AtTim21. Interactions were also tested against the carrier protein translocase of the inner membrane, AtTim22, with only AtTim21-like 2 exhibiting positive colonies. The Complex I subunit, AtB14.7, showed positive interactions with both AtTim21-like 2 and AtTim21, whilst Complex III subunit ubiquinol-cytochrome c reductase cytochrome c1 subunit (AtCyc1-1), AtTim21 also exhibited positive interaction with Complex III subunits MPPα and rieske iron sulphur protein (RISP). AtTim21 was also seen to interact with the outer membrane complex subunits AtTom9 (Tom22 in yeast) and AtTom5 (Figure 5). No interactions were observed using AtTim21-like 1, AtTim21-like 2, and AtTim21 against the pGAD empty vector confirming positive interactions.

## Discussion

In addition to Tim21, Arabidopsis mitochondria contain unique Tim21-like proteins. Whilst evolutionary distinct from each other, both contain the conserved Tim21 protein domain and phylogenetic analysis suggests that these Tim21-like proteins are plant specific. Tim21-like proteins most likely arose early in land plant evolution, as they can be identified in green algae, moss, and Selaginella. Both Tim21 and Tim21-like genes were conserved throughout all plants analyzed, containing at least one copy of each gene, suggesting that these proteins may have unique functions specific to plants.

For both AtTim21-like 1 and AtTim21-like 2, mitochondrial localization was determined by *in vitro* import assays and GFP targeting. GFP localizations showed distinct mitochondrial targeting. They were both determined to contain N-terminal targeting and assembled within the inner mitochondrial membrane. As no shift was observed following protease treatment of ruptured mitochondria we concluded that both AtTim21-like 1 and 2 are integrally located within the inner membrane. The radiolabeled band intensity of the mature protein is somewhat weak, though this is due to the fact that the native forms of AtTim21-like 1 and AtTim21-like 2, were cloned with three additional methionine's at the N-terminus for *in vitro* transcription and translation. Therefore, removal of the targeting peptide upon import results in a lower intensity band.

Import of AtTim21-like 1 and 2 and analysis via BN-PAGE revealed that these proteins may associate in the monomeric forms of Complex I and Complex III in addition to the supercomplex Complex I and III. Furthermore, AtTim21-like 2 was seen to incorporated within the Tim17:23 complex with these interactions further supported by yeast 2-Hybrid assays. This is in parallel with the previously observed interaction of AtTim21 (Wang et al., 2012) and yeast Tim21 (Van der Laan et al., 2006). AtTim21, was shown to similarly associate within Complex III using both radiolabeled proteins and immunodetection whilst interaction with the TIM17:23 complex was identified via protein interactions (Wang et al., 2012). This suggests that AtTim21 and AtTim21-like proteins may also play a role in tethering the import and respiratory chain complexes as with yeast Tim21 (Van der Laan et al., 2006). These associations are also supported by previous work involving the characterization of a AtTim21 overexpressing line, that showed alterations to mitochondrial activity and more importantly exhibited changes to the expression levels of respiratory complex subunits (Hamasaki et al., 2012).

AtTim21-like 1 and AtTim21-like 2 were also seen to associate into smaller unknown protein complexes, which remain to be identified. Although recent data showing that yeast Tim21 has the ability to associate with respiratory chain intermediates, termed MITRAC (mitochondrial protein translocation to respiratory chain assembly) containing several structural components of Complex IV plus the assembly factors COX15 and COX16 (Mick et al., 2012). In our studies weak labeling of both Complex I and Complex III controls could also be observed within these unknown complexes and thus raises the possibility that these additional complex bands observed via BN-PAGE analysis may in fact be respiratory complex assembly intermediates. Differences were also observed within the positions of these possible intermediates between AtTIm21 and AtTim21-like proteins. These small differences observed may indicate that there are some variances to protein specificity between the AtTim21 and AtTim21-like proteins and raises the possibility that AtTim21-like may interact with plant specific subunits of the respiratory complexes.

Further investigations on AtTim21 and AtTim21-like proteins will be required to determine their potential role in the assembly of respiratory complex intermediates in Arabidopsis mitochondria and to unravel the molecular mechanisms of these novel plant specific proteins involved in mitochondrial biogenesis.

## Conclusion

Here we present the mitochondrial localization of two novel Tim21-like proteins from *Arabidopsis thaliana* and show that Tim21-like 1 and 2 have the ability to interact with the translocase of the inner membrane TIM17:23 and Complex I and III of the respiratory chain. These observed interactions suggest that as seen with Tim21, Tim21-like proteins may also be involved in the import and biogenesis of respiratory chain components.

## Author contributions

Monika W. Murcha and James Whelan designed the experiments and carried out the data analysis, Monika W. Murcha, Yan Wang, and Szymon Kubiszewski-Jakubiak performed the experimental procedures and all authors contributed to the writing of the manuscript.

## Conflict of interest statement

The authors declare that the research was conducted in the absence of any commercial or financial relationships that could be construed as a potential conflict of interest.

## Supplementary material

Supplemental Table 1 **Primer pairs used for cloning**.
