# Supplementary material for: Evidence for interactions between the mitochondrial import apparatus and respiratory chain complexes via Tim21-like proteins in Arabidopsis
Source: Front Plant Sci. 2014 Mar 11;5:82. doi: 10.3389/fpls.2014.00082 (PMC3949100; doi:10.3389/fpls.2014.00082)
Supplement: Supplemental Table 1 — Primer pairs used for cloning. [file Presentation1.PDF]

Supplemental Table 1. Primer pairs used for cloning

name	AGI	details	Primer pairs
AtTim21-like 1	At2g40800	for gateway	F:GGGGACAAGTTTGTACAAAAAAGCAGGCTTCGAAGGAGATAGAACCATGATGATGGTGAAACCATCGGATTT<br>R:GGGGACCACTTTGTACAAGAAAGCTGGGTCTCCACCTCCGGATCMTGAGCCCTCCTTCTCGAG
AtTim21-like 2	At3g56430	for gateway	F:GGGGACAAGTTTGTACAAAAAAGCAGGCTTCGAAGGAGATAGAACCATGATGATGGTGAAACCTTCCGATTTC<br>R:GGGGACCACTTTGTACAAGAAAGCTGGGTCTCCACCTCCGGATCMTTTTTCAAGCTTCTCGATTTCT
AtTim21-like 1	At2g40800	Y2H	F: GGGCCGAATTCATGGTGAAAC CATCGGATTT<br>R: CCCGGGGATCCTTATGAGCCCTCCTTCTCGAG
AtTim21-like 2	At3g56430	Y2H	F: GGGCCGAATTCATGGTGAAACCTTCCGATTTC<br>R: CCCGGGGATCCCTATTTTTCAAGCTTCTCGATTTC
AtTim44-2	At2g36070	for gateway	F:GGGGACAAGTTTGTACAAAAAAGCAGGCTTCGAAGGAGATAGAACCATGGCGAGTAGAAAGC<br>R:GGGGACCACTTTGTACAAGAAAGCTGGGTCTCCACCTCCGGATCMAATGAGAGCTTGAACAC
AtTim50	At1g55900	for gateway	F:GGGGACAAGTTTGTACAAAAAAGCAGGCTTCGAAGGAGATAGAACCATGGCGTCTATTGTTCTTCG<br>R:GGGGACCACTTTGTACAAGAAAGCTGGGTCTCCACCTCCGGATCMACGCCTCCAGAATCGACC
AtTim44-2	At5g11690	Y2H	F: GGGCCGAATTCATGGCGAGTAGAAAGC<br>R: CCCGGGGATCCTTAAATGAGAGCTTGAACAC
AtTim50	At1g55900	Y2H	F: GGGCCGAATTCATGGCGTCTATTGTTCTTCG<br>R: CCCGGGGATCCTTAACGCCTCCAGAATCGACC
AtTim22	At3g10110<br>At1g18320	Y2H	F: GGGCCGAATTCATGGCTGATTCGAGTGC<br>R: CCCGGGGATCCTTATGTATGCCTATC
AtTom5	At5g08040	Y2H	F: GGGCCGAATTCATGGTGAACAACG<br>R: CCCGGGGATCCTCAAACTCCCATGAG
AtTom9	At5g43970	Y2H	F: GGGCCGAATTCATGGCGGCGAAGAGAA<br>R: CCCGGGGATCCTTACAATCCCCTTTG
AtRISP	At5g13430	Y2H	F: GGGCCGAATTCATGCTGCGTGTAGCAGGGAG<br>R: CCCGGGGATCCTCAACCGATGAGTAACTTA
At Cytochrome bd	At4g32470	for gateway	F:GGGGACAAGTTTGTACAAAAAAGCAGGCTTCGAAGGAGATAGAACCATGGCGTCGCTTCTGAAAGC<br>R:GGGGACCACTTTGTACAAGAAAGCTGGGTCTCCACCTCCGGATCMTGGGAGTGTGCGCTGG
AtCAL1	At5g63510	for gateway	F:GGGGACAAGTTTGTACAAAAAAGCAGGCTTCGAAGGAGATAGAACCATGGCGACTTCGATAG<br>R:GGGGACCACTTTGTACAAGAAAGCTGGGTCTCCACCTCCGGATCMAACGGCGATCCCAAGG
